# Iron-folic acid adherence and associated factors among pregnant women attending antenatal care at Metema District, Northwest Ethiopia

**DOI:** 10.3389/fpubh.2022.978084

**Published:** 2022-11-18

**Authors:** Ayenew Engida Yismaw, Helen Bekele Tulu, Fisseha Yetwale Kassie, Bilen Mekonnen Araya

**Affiliations:** ^1^Department of Clinical Midwifery School of Midwifery, College of Medicine and Health Science, University of Gondar, Gondar, Ethiopia; ^2^Maternity Case Team, Metema General Hospital, Amhara Regional Health Bureau, Bahir Dar, Ethiopia

**Keywords:** iron-folic acid, pregnant women, adherence, anemia, metema

## Abstract

**Background:**

Iron deficiency accounts for about half of anemia cases worldwide. Iron and folate supplementation can effectively control and prevent anemia during pregnancy. Despite the efforts to reduce iron deficiency anemia during pregnancy, only a few women took an iron supplement as recommended. The aim of this study is to assess adherence to iron-folic acid supplementation and associated factors.

**Methods:**

A facility-based cross-sectional study was conducted from April 1 to May 31, 2021, among pregnant women attending the antenatal care clinic at Metema district governmental health institutions. Data was collected through face-to-face interviews with an interviewer-administered questionnaire. Epi info version 7.1 and SPSS 20 were used for data entry and analysis. Binary logistic regression analysis was done to identify factors associated with iron-folic acid supplementation for pregnant women. Significant associations were determined based on the adjusted odds ratio (AOR) and 95% confidence, with a *p*-value < 0.05.

**Results:**

The proportion of pregnant women adhering to iron-folic acid supplementation was 34.9% (95% CI: 31, 38.8%). Maternal educational status [AOR = 6.09, 95% CI (3.26, 11.4)], time of first antenatal consultation [AOR = 1.95, 95% CI (1.25, 3.06)], having good knowledge of iron with folic acid supplementation [AOR = 2.80, 95% CI (1.83, 4.28)], having a good understanding of anemia [AOR = 1.61, 95% CI (1.06, 2.43)], and a history of anemia during current pregnancy [AOR = 2.31, 95% CI (1.36, 3.94)] were factors affecting iron-folic acid supplementation adherence.

**Conclusions:**

Iron-folic acid supplementation adherence was low in the study area. Increasing maternal awareness, having good knowledge about the benefits of iron-folate supplements, and early registration to antenatal care were positive determinants of iron with folic acid adherence.

## Introduction

Iron with folic acid (IFA) adherence measures how closely clients comply with the dosage and timing of iron-folate supplements as advised by their healthcare providers (1). If a woman takes 65% or more of the iron-folic acid supplement, which equates to taking the supplement at least 4 days a week, she is considered adherent to the supplement (2).

Iron deficiency anemia is a disorder where the body produces fewer red blood cells due to a lack of iron. A necessary nutrient for the production of hemoglobin is iron (1). Anemia occurs when the blood's hemoglobin concentration is lower than usual due to a lack of one or more crucial nutrients (3). Anemia is pervasive in public health issues and linked to a higher risk of morbidity and mortality, particularly in young children and pregnant women. More than 2 billion people, or over 30% of the world's population, suffer from iron insufficiency, the most common nutrient shortage in the world, especially in developing nations (4). Due to their increased nutritional needs, pregnant women are especially at risk of iron deficiency. Anemia caused by iron deficiency has consequences on both mother and child health. Folic acid deficiency during conception and early pregnancy causes a higher risk of neural tube abnormalities and other poor pregnancy outcomes like orofacial clefts (5).

According to the Ethiopian Demographic and Health Survey (EDHS) 2016, 23% of Ethiopian women between the ages of 15 and 49 are anemic. One-seventh of the population (15%) is slightly anemic, 5% are seriously anemic, and < 1% are severely anemic. In rural settings, 25% of women have some form of anemia, compared to 16% in urban areas (6). Since it is hard to meet the need for iron during pregnancy solely by dietary intake, iron-folic acid supplementation is a widely advised intervention to address this problem (7).

Therefore, giving iron and folic acid in the form of tablets to pregnant women is the most effective mass intervention for iron supplementation. These increase hemoglobin levels, allowing for the possible reduction in the level of anemia at term (8).

All pregnant women have been advised to take a daily supplement containing 400 μg of folic acid and 30–60 mg of elemental iron for 6 months by the World Health Organization. Supplemental iron and folic acid should be used with malaria prevention, detection, and treatment strategies (9).

The main issue with iron-folate supplementation during pregnancy is adherence because women frequently forget to take the supplements regularly as directed by their healthcare providers. This is the probable cause of the high prevalence of anemia among pregnant mothers.

## Methods

### Study design and setting

A facility-based cross-sectional study was conducted in Metema district governmental health institutions located in the West Gondar Zone of the Amhara Regional State, Ethiopia, from April 1 to May 31, 2021. Metema district is 897 km north of Addis Ababa and 197 km from Gondar town. The district has one general hospital and five health centers.

### Sample size determination and sampling technique

The sample size was calculated by single population proportion formula using a 95% confidence level, a 5% margin of error, a prevalence of 37.2% (5), a design effect of 1.5, and a non-response rate of 10%. Four health centers (Gendawuha, Kokit, Metema Yohannes, and Shinfa) were selected randomly for the study by lottery method. Study participants were selected using a systematic and simple random sampling method. Reports of the 2 months of the study period were taken from each selected health institution to allocate the sample proportionally. The total number of pregnant women attending the antenatal care (ANC) clinic was 1,318. The required minimum sample size in the institutions was *n* = 593; an interval of *k* = N/n = 1,318/593 = 2 was used to select the study participants. The first study participant was selected using the lottery method and then every other woman was included. All pregnant women attending routine antenatal care follow-up for the current pregnancy and who took IFA supplementation (IFAS) at least for a month during the study period were the study population. All pregnant women who took IFAS at least for a month and had ANC follow-ups at Metema district governmental health centers were included in the study. Adherence to IFAS was defined as pregnant women taking IFAS tablets at least four times per week in the previous month preceding the study (2, 5, 8).

### Data collection method

Face-to-face interviews were used to gather data using a pretested questionnaire. The questionnaire was prepared after reviewing the literature (3–7, 9). To make the questionnaires clear, it was first prepared in English and translated into the local (Amharic) language to make them simple and understandable, and then back to English to check the consistency. Five percent of the sample size in Metema primary hospital pretested the questionnaire, based on which the questionnaire underwent necessary corrections.

Four BSc nurses and two supervisors collected the data. The data collectors and supervisors received a one-day training. The pregnant women were interviewed before they got their regular service/examination.

### Statistical analysis

Epi-info version 7.1 software was used to enter the data which was exported into SPSS version 20 software for statistical analysis and interpretation. The sociodemographic variables of pregnant women were analyzed with descriptive statistics. Binary logistic regression analysis was used to determine the association of independent variables with IFAS as a dependent variable. Variables that were significant in binary logistic regression with a *p*-value < 0.25 were considered for multivariable logistic regression analysis. To demonstrate the strength of the association, crude odds ratio (COR) and adjusted odds ratio (AOR), together with the 95 percent confidence interval, were calculated. Variables having a *p*-value of 0.05 or lower were deemed statistically significant in the multivariable logistic regression analysis.

### Ethical consideration

The institutional review board (IRB) of the University of Gondar College of Medicine and Health Sciences granted ethical approval with the reference number (SMidw/53/2013). A permission letter was also obtained from the Metema district health office. Anonymity was used to protect the confidentiality of the study participant's data. Written informed consent was taken from the study participants. The data was not used for any other purpose other than the designed study.

## Results

### Sociodemographic characteristics of pregnant women

A total of 575 pregnant mothers took part in the study yielding a 97% response rate. The average age of the pregnant women was 27.1 with (SD) ±5.57 years old. More than half of the participants 300 (52.2%) were in the age group of 25–34 years. About 367 (63.8%) were Orthodox Christian followers and two-thirds (383, 66.6%) were urban residents. About 322 (56%) of the mothers had >1,000 birr monthly income (Table 1).

**Table 1:** Socio-demographic characteristics of pregnant women attending ANC clinic in governmental health institutions of Metema district, Northwest Ethiopia, May, 2021 (*N* = 575).

**Variable**	**Category**	**Frequency**	**Percentage**
Age	15–24 years	202	(35.1)
	25–34 years	300	(52.2)
	35–49 years	73	(12.7)
Educational level	Can't read and write	145	(25.2)
	Read & write only	126	(21.9)
	Primary	144	(25.1)
	Secondary and above	160	(27.8)
Marital status	Married	534	(92.9)
	Un married	41	(7.1)
Occupational status	House wife	364	63.3
	Government employee	131	(22.8)
	Daily laborer	9	(1.6)
	Merchant	71	(12.3)
Family size	1–3	326	(56.7)
	4–6	202	(35.1)
	>6	47	(8.2)
Husband's Education	Can't read and write	171	(32)
	Can read and write	145	(27.2)
	Primary	56	(10.5)
	Secondary and above	162	(30.3)

### Obstetric and health facility-related characteristics of pregnant women

The average gestational age of participants during the interview was 26.1 ±5.3 weeks. About 324 (56.3%) participants were in their second trimester. About two-fifths (229, 39.8%) of participants were primigravida. A majority (345, 60%) had a history of anemia, and an equal number t 340 (59.1%) visited the ANC clinic within 12 weeks of gestation. About two-thirds (393, 68.3%) of the respondents spent < 30 min reaching the health facility.

### Adherence rate to IFAS

The adherence rate to IFA supplementation was 34.9% (95%CI, 31.0–38.8%). The leading reason for IFAS adherence was counseling (65.6%), followed by fear of illness if they missed the supplement (21.9%), and finally, family support (17.2%). Among mothers who missed the IFAS doses, the leading reason was fear of side effects (83.3%) followed by forgetfulness (40.9%).

### Factors associated with adherence to IFA supplementation

Maternal education, time of ANC registration, history of anemia, the prevalence of anemia during the current pregnancy, counseling on IFA, knowledge of anemia, and knowledge of IFAS were associated with IFAS adherence in bivariable analysis at *p*-value < 0.25 and entered multivariable logistic regression analysis. In the multivariate logistic regression analysis, there were significant relationships between IFAS adherence and the timing of ANC registration, the prevalence of anemia during the current pregnancy, maternal education, awareness of anemia, and knowledge of IFAS.

The odds of pregnant mothers with a secondary level school education and above were six times more likely to adhere to IFAS than those who cannot read and write [AOR = 6.09 95% CI (3.26, 11.4)]. The odds of pregnant women registering earlier for ANC were 1.9 times more likely to adhere to IFAS than those who registered late [AOR = 1.95, 95% CI (1.25, 3.06)]. The odds of pregnant women who were knowledgeable about anemia were 1.6 times more likely to adhere to IFAS than those who were less knowledgeable [AOR = 1.61, 95% CI (1.06, 2.43)]. Pregnant women who were knowledgeable about IFAS were 2.8 times more likely to adhere to IFAS than those less knowledgeable about IFAS [AOR = 2.80, 95% CI (1.83, 4.28)]. The odds of pregnant women who had a history of anemia during current pregnancy were 2.3 times more likely to adhere to IFAS than those who had no anemia [AOR = 2.31, 95% CI (1.36, 3.94)] (Table 2).

**Table 2:** Factors associated with adherence to IFAS among pregnant women attending ANC Clinics at Metema woreda governmental health institutions, North west Ethiopia, May 2021 (*N* = 575).

**Variables**	**Adherence**			
	**Yes**	**No**	**COR(95%CI)**	**AOR(95%CI)**
**Educational status**				
Can't read and write	22	123	1	1
Read and write only	40	86	2.60 (1.44, 4.69)	2.58 (1.34, 4.94)^**^
Primary education	57	87	3.66 (1.09, 6.43)	2.07 (1.64, 5.75)^**^
Secondary and above	82	78	5.88 (3.39, 10.18)	6.09 (3.26, 11.4)^**^
**Marital Status**				
Married	339	195	3.36 (1.39, 8.12)	2.59 (0.992, 6.75)
Un married	35	6	1	1
**Time of ANC registration**				
Early(< 12 weeks)	129	111	2.34 (1.65, 3.325)	1.95 (1.25, 3.06)^**^
Late (≥12weeks)	245	90	1	1
**Knowledge of IFAS**				
Knowledgeable	185	146	2.71 (1.87, 3.93)	2.80 (1.83, 4.28)^**^
Less knowledgeable	189	55	1	1
**Knowledge of Anemia**				
Knowledgeable	115	103	2.37 (1.66, 3.37)	1.61 (1.06, 2.43)^**^
Less knowledgeable	259	98	1	1
**History of previous anemia**				
No	67	63	1	1
Yes	134	211	1.54 (1.08, 2.21)	1.05 (0.68, 1.61)
**Counseling on IFAS**				
No	56	142	1	1
Yes	145	232	1.58 (1.09, 2.30)	1.47 (0.92, 2.35)
**History of anemia in current pregnancy**				
No	26	97	1	1
Yes	175	277	2.36 (1.47, 3.78)	2.31 (1.36, 3.94)^**^

** variables associated in multivariate analysis and statistically significant at *p* < 0.05.

## Discussion

Iron-folic acid supplementation adherence among pregnant mothers attending ANC clinics was 34.9% (95% CI 31.0–38.8%). The finding of this study is consistent with the findings in Tigray, Ethiopia (37.2%) (5). This might be due to the similarities in the study population, settings, and service delivery of the institutions.

However, it is higher compared with the studies done in Mecha district, Northwest Ethiopia (20.4%) (10), Afar region (22.9%) (11), Goba woreda (18%) (12), Bihar, India (24%) (13), Moshi, Tanzania (16.1%) (14) and Kiambu county, Kenya (24.5%) (15). The reason might be increased knowledge among pregnant mothers over time regarding anemia and IFA supplementation through health education, counseling, media, improved ANC service coverage, and the time difference between studies.

However, the adherence levers were lower than the studies done in Mizan Aman town (70.6%) (16), Misha district, South Ethiopia (39.2%) (17), eight rural districts of Ethiopia (74.9%) (18), Siem Reap and Kampong Cham Provinces, Cambodia (47%) (19), Indonesia (53.7%) (20), and Mangalore city, India (64.7%) (8). The variation may be dissimilarity in the design of the studies done in Misha district, South Ethiopia, and eight rural districts of Ethiopia which were community-based. The others could be explained by differences in health service delivery and the socio-economic status of study populations for the studies done in Cambodia, Indonesia, and India.

Iron-folic acid adherence was better observed among pregnant mothers who had attended secondary school and above. This finding is similar to other findings in urban slums in India, and Mecham district, Northwest Ethiopia (7, 10, 21). The reason behind this might be that educated women have good knowledge regarding the benefit of IFAS for their health and the health of the fetus. Plus, educated women have better knowledge of anemia and its effect on the health of the mother and their fetus.

Early registration for ANC services had a better effect on IFAS adherence among pregnant mothers than those who registered late. This finding is similar to several other studies done in urban slums in India, Tigray, and other areas in Ethiopia (5, 13, 21, 22). The reason may be pregnant women who had early registration for ANC services probably had more ANC visits leading to better counseling and ultimately enhancing their knowledge about anemia and IFAS.

The prevalence of anemia during the current pregnancy was another factor that increased adherence to IFAS. This finding is consistent with other studies on Mecha district, Tigray, Kiambu county, Kenya, and Moshi, Tanzania (5, 10, 14, 15). This might be due to the attention given to counseling anemic women, which increased their awareness and knowledge of iron and folic acid supplementation, and therefore they took the supplements regularly to avoid complications.

Having good knowledge about iron-folic acid supplementation led mothers to take the tablets regularly. This finding is similar to other studies done in Siem Reap and Kampong Cham Provinces in Cambodia, urban slums in India, Misha district of Ethiopia, and Mecha (10, 17, 19, 21). The possible reason is that being knowledgeable about IFAS made mothers more aware of the tablet's importance, its side effects, how to take them, and complications if missed. Being knowledgeable about anemia made the mothers adhere to iron-folic acid supplementation. A similar result was reported in Mecha district, Western Amhara (10). The probable reason could be knowledge of pregnant women about causes, consequences, and methods of prevention of anemia affected their adherence to IFA. In addition, being knowledgeable about iron and folic acid encourages individuals to prevent iron deficiency anemia and helps women to adopt a good attitude about the benefits of taking IFA supplements.

## Conclusion

This study found that IFA supplementation adherence among pregnant mothers attending antenatal care is low. This study also found that maternal education, knowledge of IFAS, history of anemia in the current pregnancy, time of ANC registration, and knowledge of anemia were significant factors that were associated with adherence to IFAS during pregnancy. Therefore, antenatal care providers should continue to advise pregnant mothers to register earlier for ANC and counsel them on the importance of IFAS. Also, the district health offices should promote IFAS and anemia prevention strategies in the community through community health task forces.

## Limitations

Electronic pill counting which, is the gold standard method of measuring adherence had not been used as it is expensive. Being cross-sectional, this study might not show cause-effect relations. It was also important if the nutritional assessment had been included.

## Data availability statement

The raw data supporting the conclusions of this article will be made available by the authors, without undue reservation.

## Ethics statement

The studies involving human participants were reviewed and approved by the Institutional Review Board of the University of Gondar College of Medicine and Health Sciences. The patients/participants provided their written informed consent to participate in this study.

## Author contributions

AY conceived and designed the experiments. HT, FK, and BA contributed in investigation, analyzed and interpreted the data, and wrote the article. All authors contributed to the article and approved the submitted version.

## Conflict of interest

The authors declare that the research was conducted in the absence of any commercial or financial relationships that could be construed as a potential conflict of interest.

## Publisher's note

All claims expressed in this article are solely those of the authors and do not necessarily represent those of their affiliated organizations, or those of the publisher, the editors and the reviewers. Any product that may be evaluated in this article, or claim that may be made by its manufacturer, is not guaranteed or endorsed by the publisher.

## Abbreviations

ANC: Antenatal Care
AOR: Adjusted Odds Ratio
CI: Confidence interval
COR: Crude Odds Ratio
IFA: Iron Folate Acid
IFAS: Iron Folate Acid Supplimentaion
SPSS: Statistical Package for Social Science.
